# Supplementary material for: Identification of Freezing-Responsive microRNAs and Their Targets in Chinese Jujube by Small RNA and Degradome Sequencing
Source: Int J Mol Sci. 2026 Sep 4;27(17):7912. doi: 10.3390/ijms27177912 (PMC13566890; doi:10.3390/ijms27177912)
Supplement: Supplementary file 1 [file ijms-27-07912-s001.zip › Supplementary Material S2/novel_miR_124.pdf]

Provisional ID : novel_miR_124
Score total : 1013.6
Score for star read(s) : 3.9
Score for read counts : 1007.5
Score for mfe : 0.6
Score for randfold : 1.6
Score for cons. seed :
Total read count : 1988
Mature read count : 1973
Loop read count : 0
Star read count : 15

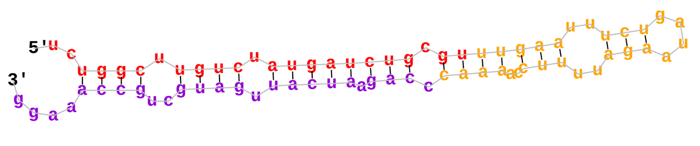

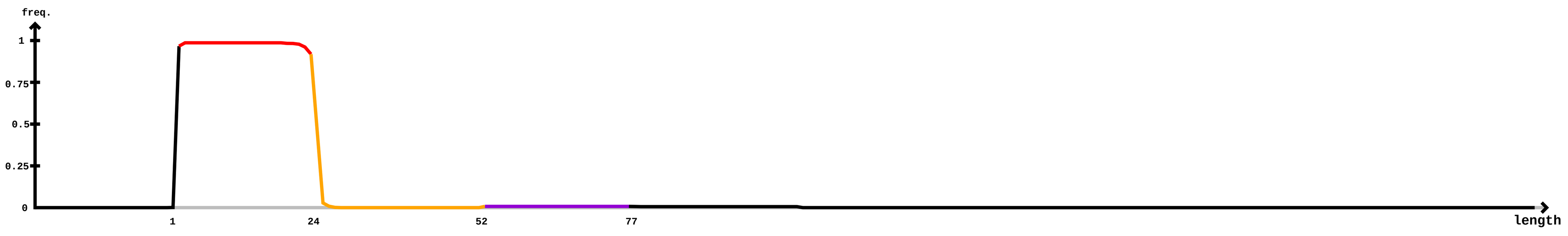


```
                      Mature                                                 Star
5'- uaugguuagcaucuucucagccucuggcuugucuaugaucugcguuugaauuucugauaagauuuuccaaaacccagaaucauugaugcugccaaaggcuggaaugacaaucucuuuuaaguaacuucccuugcuuuuuugguucuuuucuugaaccggcaucuaaagauugauggaguucugauucuugauaaccauaacuaccucuuuucuuuuucucuagaugcuuuguucugaugcugcauuugag  -3'  obs
    uaugguuagcaucuucucagccucuggcuugucuaugaucugcguuugaauuucugauaagauuuuccaaaacccagaaucauugaugcugccaaaggcuggaaugacaaucucuuuuaaguaacuucccuugcuuuuuugguucuuuucuugaaccggcaucuaaagauugauggaguucugauucuugauaaccauaacuaccucuuuucuuuuucucuagaugcuuuguucugaugcugcauuugag       exp
    ....((.(((((((((.(((((.((((.((((.((((((((.(((((((..(((....)))..)))..)))).))).))))).)))).)))).)))))))))............((((((......))))))....(((((.......)))))((((((((.(((..((.((((((.((...........)).)))).)))).........))).))))))))......))))))).......   reads   mm   sample
    .....................cucuggcuugucuaugaucugcgu.............................................................................................................................................................................................................   2   0   JCK-2
    ......................ucuggcuugucuaugauc..................................................................................................................................................................................................................   1   0   JCK-2
    ......................ucuggcuugucuaugauc..................................................................................................................................................................................................................   1   0   JCK-1
    ......................ucuggcuugucuaugauc..................................................................................................................................................................................................................   4   0   LCK-3
    ......................ucuggcuugucuaugauc..................................................................................................................................................................................................................   1   0   JCK-3
    ......................ucuggcuugucuaugaucu.................................................................................................................................................................................................................   1   0   LT-1
    ......................ucuggcuugucuaugaucug................................................................................................................................................................................................................   3   0   JCK-2
    ......................ucuggcuugucuaugaucug................................................................................................................................................................................................................   3   0   JCK-3
    ......................ucuggcuugucuaugaucug................................................................................................................................................................................................................   2   0   JCK-1
    ......................ucuggcuugucuaugaucug................................................................................................................................................................................................................   1   0   LT-3
    ......................ucuggcuugucuaugaucugc...............................................................................................................................................................................................................   5   0   LCK-3
    ......................ucuggcuugucuaugaucugc...............................................................................................................................................................................................................   4   0   JCK-1
    ......................ucuggcuugucuaugaucugc...............................................................................................................................................................................................................   8   0   JCK-3
    ......................ucuggcuugucuaugaucugc...............................................................................................................................................................................................................   4   0   LT-3
    ......................ucuggcuugucuaugaucugc...............................................................................................................................................................................................................   13  0   JCK-2
    ......................ucuggcuugucuaugaucugcg..............................................................................................................................................................................................................   23  0   JCK-3
    ......................ucuggcuugucuaugaucugcA..............................................................................................................................................................................................................   1   1   JCK-2
    ......................ucuggcuugucuaugaucugcg..............................................................................................................................................................................................................   1   0   LT-1
    ......................ucuggcuugucuaugaucugAg..............................................................................................................................................................................................................   1   1   JCK-2
    ......................ucuggcuugucuaugaucugcg..............................................................................................................................................................................................................   3   0   LT-2
    ......................ucuggcuugucuaugaucugcA..............................................................................................................................................................................................................   1   1   JCK-3
    ......................ucuggcuugucuaugaucugcg..............................................................................................................................................................................................................   2   0   LCK-3
    ......................ucuggcCugucuaugaucugcg..............................................................................................................................................................................................................   1   1   JCK-3
    ......................ucuggcuugucuaugaucugcg..............................................................................................................................................................................................................   32  0   JCK-2
    ......................ucuggcuugucuCugaucugcg..............................................................................................................................................................................................................   1   1   JCK-1
    ......................ucuggcuugucuaugCucugcg..............................................................................................................................................................................................................   1   1   JCK-2
    ......................ucuggcuugucuaugaucugcA..............................................................................................................................................................................................................   1   1   LT-1
    ......................ucuggcuugucuaugaucugcg..............................................................................................................................................................................................................   14  0   JCK-1
    ......................ucuggcuugucuaugaucugcg..............................................................................................................................................................................................................   3   0   LT-3
    ......................ucuggcuugucuCugaucugcgu.............................................................................................................................................................................................................   2   1   JCK-1
    ......................ucuggcuugucuaugaucugUgu.............................................................................................................................................................................................................   1   1   JCK-2
    ......................ucuggcuugucuaugaucugcAu.............................................................................................................................................................................................................   1   1   JCK-2
    ......................ucuggcuuAucuaugaucugcgu.............................................................................................................................................................................................................   1   1   LT-3
```

Mature **Star**

uaugguuagcaucuucucagccucuggcuugucuaugaucugcguuugaauuucugauaagauuuuccaaaacccagaaucauugaugcugccaaaggcuggaaugacaaucucuuuuaaguaacuucccuugcuuuuuugguucuuuucuugaaccggcaucuaaagauugauggaguucugauucuugauaaccauaacuaccucuuuucuuuuucucuagaugcuuuguucugaugcugcauuugag

ucuggcuugucuaugaucugNgu	1	1	JCK-1
ucuggcuugucCaugaucugcgu	1	1	JCK-2
ucuggcuugucuaugaucuNcgu	1	1	JCK-1
ucuggcuugucuaugaucugcgu	66	0	LT-3
Ccuggcuugucuaugaucugcgu	1	1	JCK-2
ucuggcuugucuaugaucugcgA	2	1	LCK-3
ucuggcuugucuaugaucugcgu	137	0	LCK-3
ucuggcuugucuaugaucugcgA	1	1	JCK-3
uNuggcuugucuaugaucugcgu	1	1	JCK-3
ucuggcuugucuaugaucugcgu	290	0	JCK-1
ucuggcuugucuCugaucugcgu	1	1	LCK-3
ucuggcuugucuaugaucugcgu	463	0	JCK-2
ucuggcuugucuaugaucugcgu	26	0	LT-2
ucuggcuugucuaugaucugUgu	1	1	LCK-3
ucuggcuugucuaugaucugcgA	3	1	JCK-2
ucuggcuugucuCugaucugcgu	4	1	JCK-3
ucuggcuugucuaugaucugcgu	64	0	LT-1
ucuggcuugucuaugaucugcgu	4	0	JT-2
ucuNgcuugucuaugaucugcgu	1	1	JCK-1
ucuggcuugucNaugaucugcgu	4	1	JCK-1
ucuggcuugucuaugaucugcgA	1	1	JCK-1
ucuggcuugucuaugaucugcgu	319	0	JCK-3
ucuggcuugucuaugaucugcAu	1	1	JCK-3
ucuggcuugCcuaugaucugcgu	2	1	JCK-2
ucuggcuugucuCugaucugcgu	1	1	LT-3
ucuggcuugucuCugaucugcgu	4	1	JCK-2
ucuggcuugucuaugCucugcgu	1	1	JCK-2
ucuggcuugucuaugaucugcgu	6	0	JT-3
ucuggcuugucuaugaucugcgAu	2	1	JCK-1
ucuggcuugucuaugaucugcguu	7	0	JT-3
ucuggcuugucuaugaucugcguu	17	0	LT-3
ucuggcuugucuaugaucugcguA	28	1	JCK-3
ucuggcuugucuaugaucugcguC	1	1	LT-2
ucuggcuugucuaugaucugcguA	5	1	LT-3
ucuggcuugucuaugaucugcguA	34	1	JCK-1
ucuggcuugucuaugaucugcguA	36	1	JCK-2
ucuggcuugucuCugaucugcguu	1	1	JCK-1
ucuggcuugucuaugaucugcgAu	1	1	JCK-3
ucuggcuugucuauUaucugcguu	1	1	JCK-2
ucuggcuugucuaugaucugcguA	6	1	LT-2
ucuggcuugucuaugaucugcguA	7	1	LCK-3
ucuggcuugucuaugaucugcguu	12	0	LT-2
ucuggcuugucuaugaucugcguC	2	1	LT-1
ucuggcuugucuaugaucugcguu	1	0	JT-2
ucuggcuugucuaugaucugcguu	38	0	JCK-3
ucuggcuugucuaugaucugcguu	33	0	JCK-1
ucuggcuugucuaugaucugcguu	4	0	LCK-3
ucuggcuugucuaugaucugcguu	53	0	JCK-2
ucuggcuugucuaugaucugcguu	29	0	LT-1
ucuggcuugucuCugaucugcguu	2	1	JCK-3
ucuggcuugucuaugaucugcguA	6	1	LT-1
ucuggcuugucuaugaucugcUuu	1	1	LT-1
ucuggcuugucuaugaucugcguuC	2	1	JCK-2
ucuggcuugucuaugaucugcguuu	3	0	JCK-1
ucuggcuugucuaugaucugcguuA	3	1	LT-3
ucuggcuugucuaugaucugcguAu	1	1	JCK-2
ucuggcuugucuaugaucugcguuC	3	1	LT-2
ucuggcuugucuaugaucugcguuA	2	1	JCK-2
ucuggcuugucuaugaucugcguuu	2	0	JCK-2
ucuggcuugucuaugaucugcguuC	2	1	JCK-3
ucuggcuugucuaugaucugcguuu	2	0	JCK-3
ucuggcuugucuaugaucugcguuC	1	1	JCK-1
ucuggcuugucuaugaucugcguuC	1	1	LT-3
ucuggcuugucuaugaucugcguuC	2	1	LT-1
ucuggcuugucuaugaucugcguuu	6	0	LT-1
ucuggcuugucuaugaucugcguuu	1	0	LT-3
ucuggcuugucuaugaucugcguuu	7	0	LT-2
ucuggcuugucuaugaucugcguuA	1	1	JCK-1
ucuggcuugucuaugaucugcguuuA	1	1	LT-3
ucuggcuugucuaugaucugcguuuU	3	1	LT-1

```
                      Mature                                                  Star
uaugguuagcaucuucucagccucuggcuugucuaugaucugcguuugaauuucugauaagauuuuccaaaacccagaaucauugaugcugccaaaggcuggaaugacaaucucuuuuaaguaacuucccuugcuuuuuugguucuuuucuugaaccggcaucuaaagauugauggaguucugauucuugauaaccauaacuaccucuuuucuuuuucucuagaugcuuuguucugaugcugcauuugag
......................ucuggcuugucuaugaucugcguuuA.........................................................................................................................................................................................................    2    1    LT-1
......................ucuggcuugucuaugaucugcguuuU.........................................................................................................................................................................................................    3    1    JCK-3
......................ucuggcuugucuaugaucugcguuuU.........................................................................................................................................................................................................    4    1    JCK-2
......................ucuggcuugucuaugaucugcguuuU.........................................................................................................................................................................................................    1    1    LT-3
......................ucuggcuugucuaugaucugcguuuUa........................................................................................................................................................................................................    1    1    JCK-3
......................ucuggcuugucuaugaucugcguuuUa........................................................................................................................................................................................................    2    1    JCK-2
.......................cuggcuugucuaugaucugcgu.............................................................................................................................................................................................................    2    0    LCK-3
.......................cuggcuugucuaugaucugcAu.............................................................................................................................................................................................................    1    1    JCK-2
.......................cuggcuugucuaugaucugcgu.............................................................................................................................................................................................................    5    0    JCK-3
.......................cuggcuugucuaugaucugcgu.............................................................................................................................................................................................................    5    0    JCK-1
.......................cuggcuugucuCugaucugcgu.............................................................................................................................................................................................................    1    1    LCK-3
.......................cuggcuugucuaugaucugcgu.............................................................................................................................................................................................................    15   0    JCK-2
.......................cuggcuugucuaugaucugcguA............................................................................................................................................................................................................    2    1    JCK-3
.......................cuggcuugucuCugaucugcguu............................................................................................................................................................................................................    1    1    JCK-3
.......................cuggcuugucuaugaucugcguu............................................................................................................................................................................................................    1    0    JCK-2
.......................cuggcuugucuaugaucugcguu............................................................................................................................................................................................................    1    0    JCK-3
.......................cuggcuugucuaugaucugcguA............................................................................................................................................................................................................    2    1    JCK-2
.......................cuggcuugucuaugaucugcguu............................................................................................................................................................................................................    1    0    JCK-1
.......................cuggcuugucuaugaucugcguA............................................................................................................................................................................................................    3    1    JCK-1
.........................................................................ccagaaucauugaugcugccaaagg........................................................................................................................................................    15   0    LCK-3
..................................................................................................cuggaaugacaaucucuuuuaaguaacu............................................................................................................................    4    0    LCK-2
..................................................................................................cuggaaugacaaucucuuuuaaguaacu............................................................................................................................    6    0    LCK-1
..................................................................................................cuggaaugacaaucNcuuuuaaguaacu............................................................................................................................    1    1    LCK-1
..................................................................................................cuggaaugacaaucucuuuuCaguaacu............................................................................................................................    1    1    LCK-2
```
